# Supplementary material for: SARS-CoV-2 infection risk during delivery of childhood vaccination campaigns: a modelling study
Source: medRxiv. 2021 Jul 22:2021.05.14.21257215. Preprint. [Version 3] doi: 10.1101/2021.05.14.21257215 (PMC8142667; doi:10.1101/2021.05.14.21257215)
Supplement: Supplement 1 [file media-1.pdf]

Supplementary Figures for “SARS-CoV-2 infection risk during delivery of childhood vaccination campaigns: a modelling study”

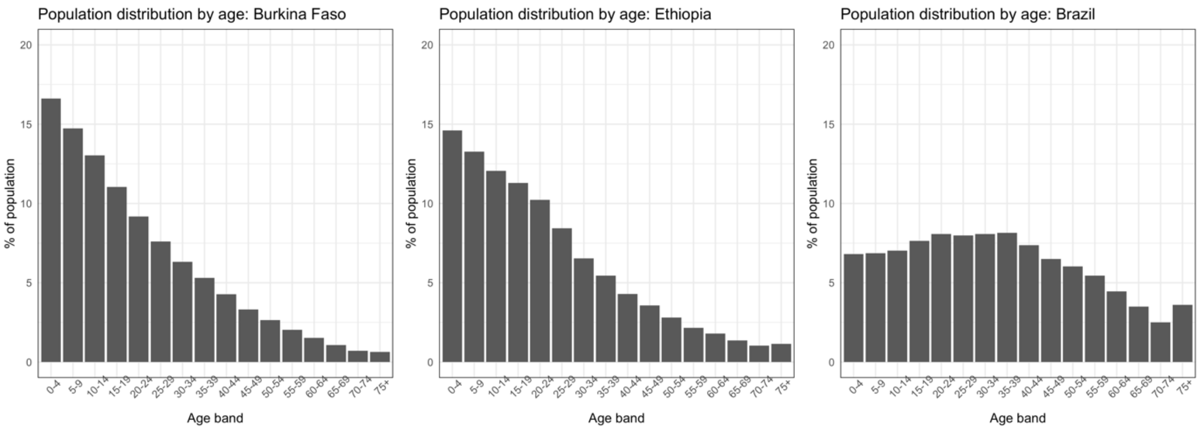

Supplementary Figure 1. Population age distribution used to parameterise the transmission model

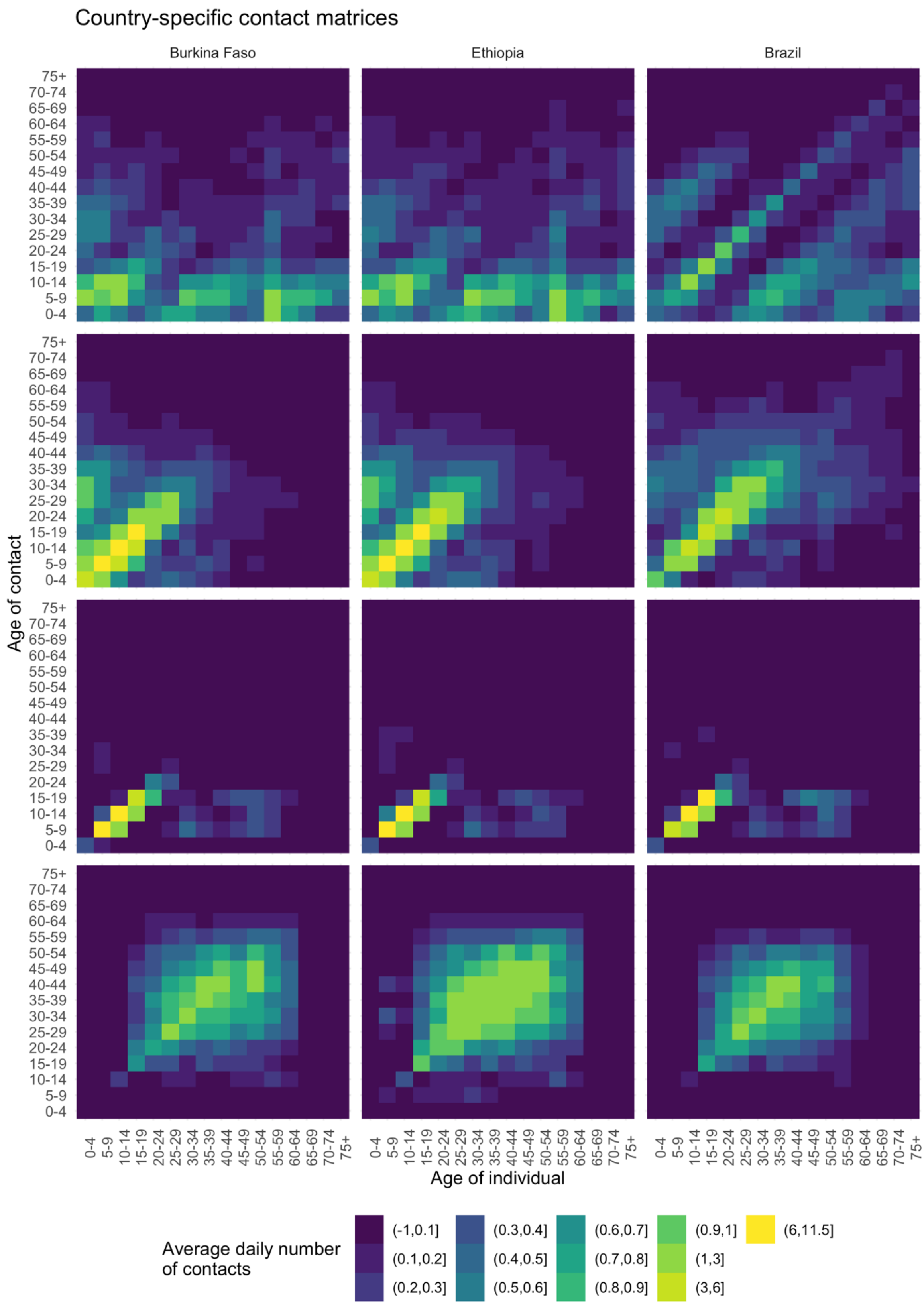

Supplementary Figure 2. Baseline age-specific contact matrices from Prem et al. (prior to assumed reduction in non-household contacts) used to parameterise the transmission model

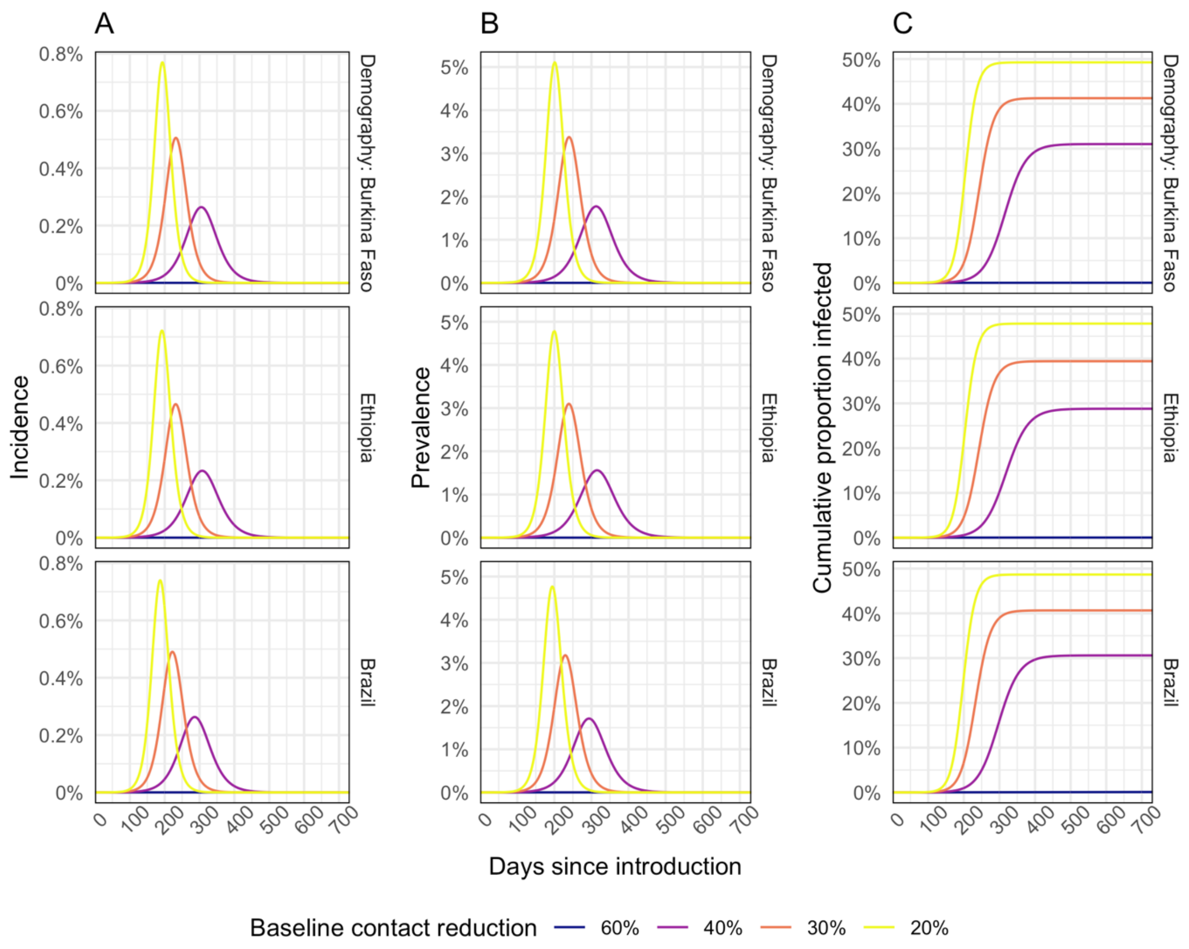

Supplementary Figure 3. Modelled incidence (A), prevalence (B) and cumulative proportion of individuals infected (C) for an epidemic with R_0 = 2 and assuming different % reductions in non-household contacts compared to baseline. These simulations are used in two different sensitivity analyses: (i) using results for 30% and 20% reductions applied to vaccinators only to model the impact of faster epidemics amongst healthcare workers (Supplementary Figure 4); and (ii) using results for 60% and 20% reductions as a sensitivity analysis applied to the whole population.

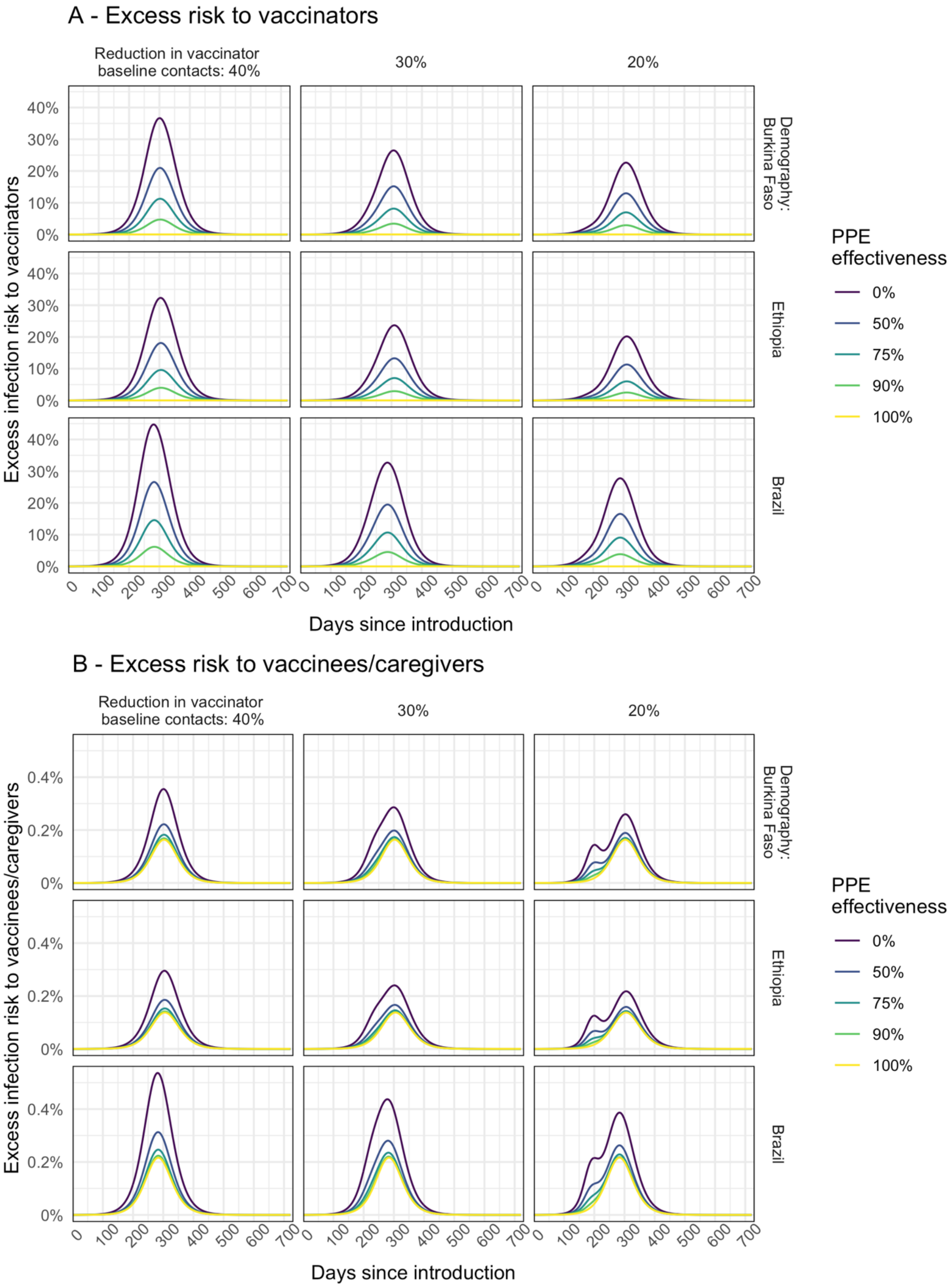

Supplementary Figure 4. Excess infection risk for vaccinators (A) and children and/or caregivers (B) for scenarios in which vaccinators are assumed to experience a smaller reduction in non-household contacts compared to the general population.

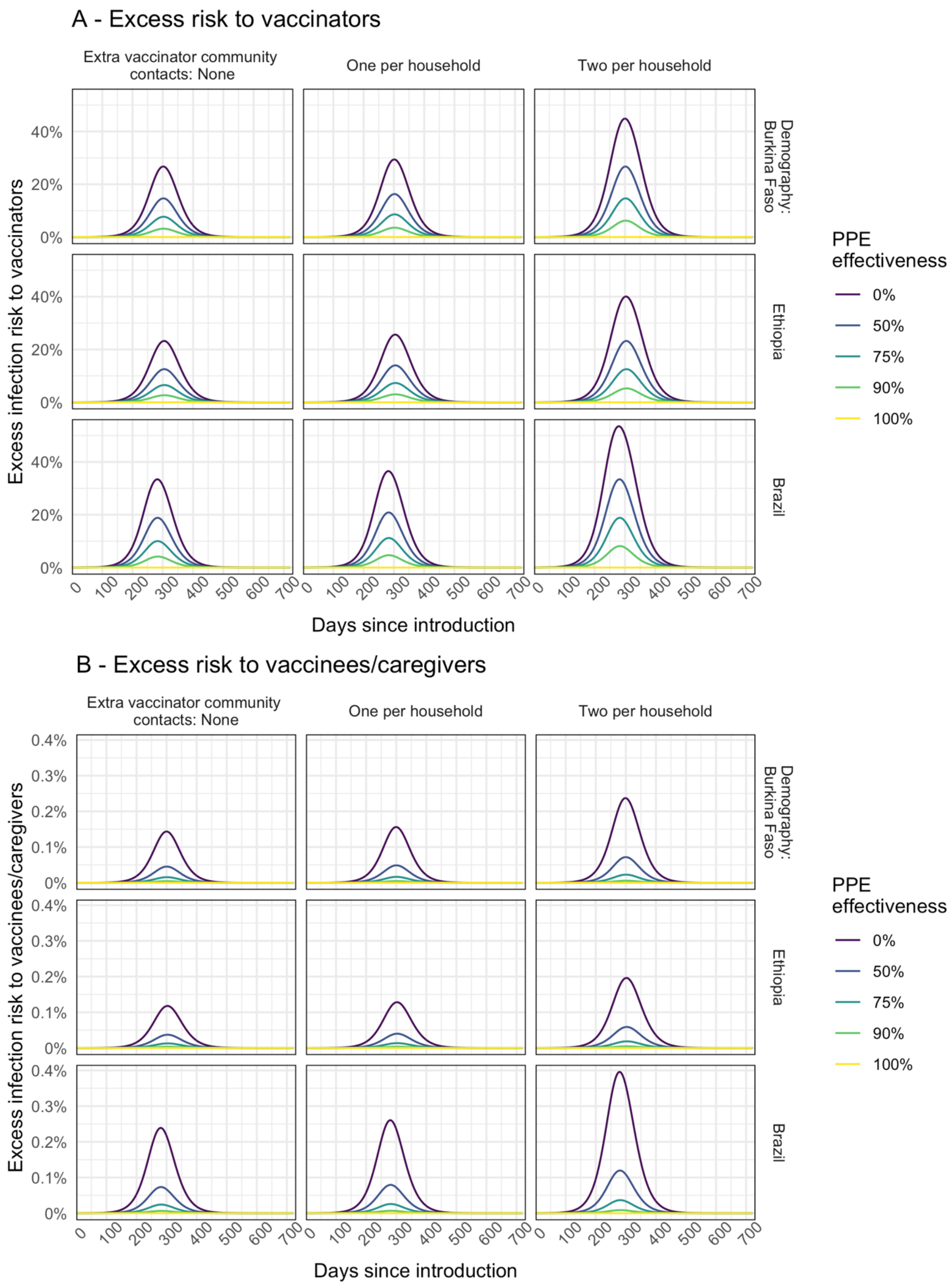

Supplementary Figure 5. Modelled excess risk of (A) vaccinators and (B) vaccinnees and/or caregivers becoming infected during house-to-house immunisation campaigns conducted at different times during the epidemic. Results are shown for epidemics modelled using R_0=2 and different assumptions about the number of additional community contacts made by vaccinators for each household visited. Line colour shows the impact of different levels of PPE effectiveness.

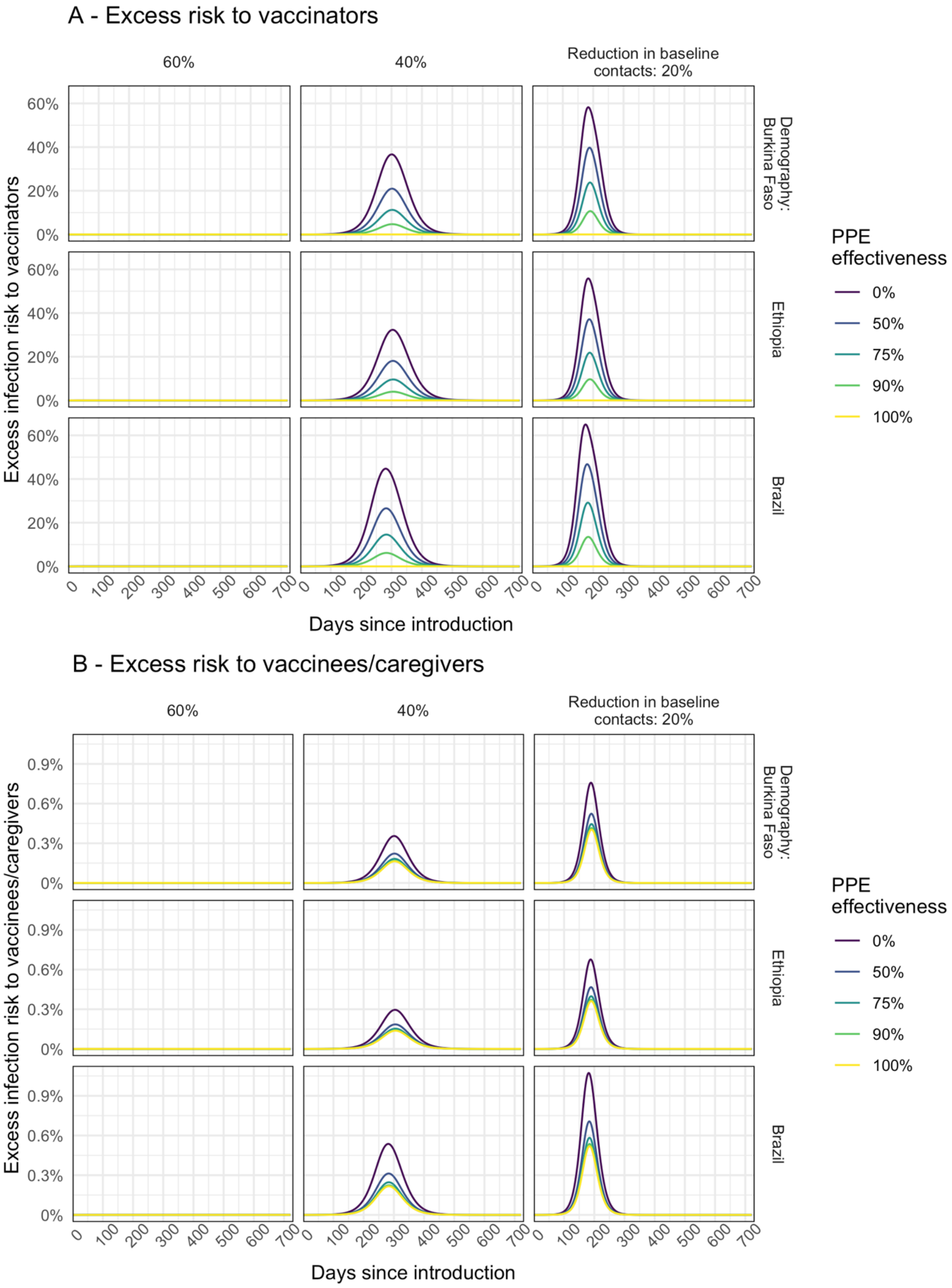

Supplementary Figure 6. Modelled excess risk of (A) vaccinators and (B) vaccinnees and/or caregivers becoming infected during fixed-post immunisation campaigns conducted at different times during the epidemic. Results are shown for epidemics modelled using R_0=2 and different assumptions about the level of reduction in non-household contacts due to NPIs. Line colour shows the impact of different levels of PPE effectiveness.
