# Supplementary material for: SARS-CoV-2 infection risk during delivery of childhood vaccination campaigns: a modelling study
Source: medRxiv. 2021 May 19:2021.05.14.21257215. Preprint. [Version 2] doi: 10.1101/2021.05.14.21257215 (PMC8142667; doi:10.1101/2021.05.14.21257215)
Supplement: Supplementary file 1 [file media-1.pdf]

Supplementary Appendix for: SARS-CoV-2 infection risk during delivery of childhood vaccination campaigns: a modelling study

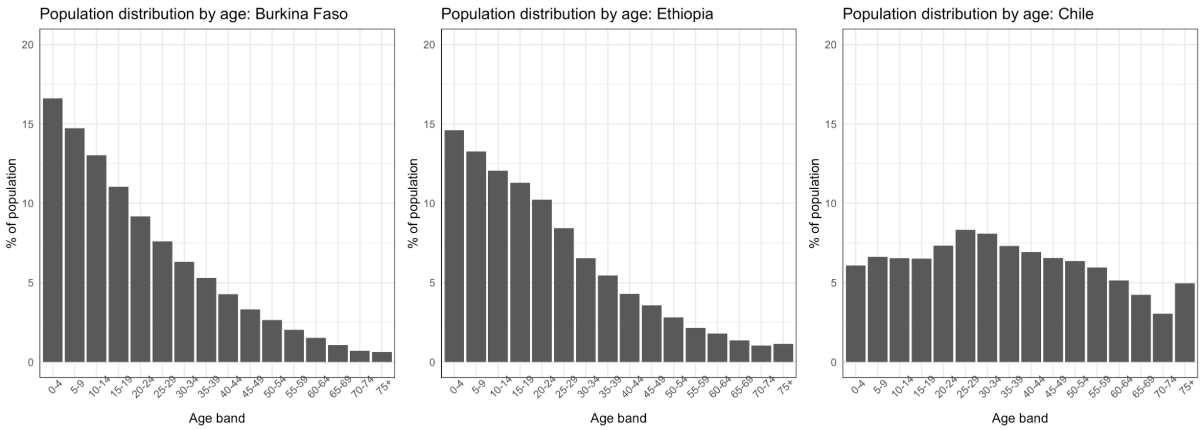

Supplementary Figure 1. Population age distribution used to parameterise the transmission model

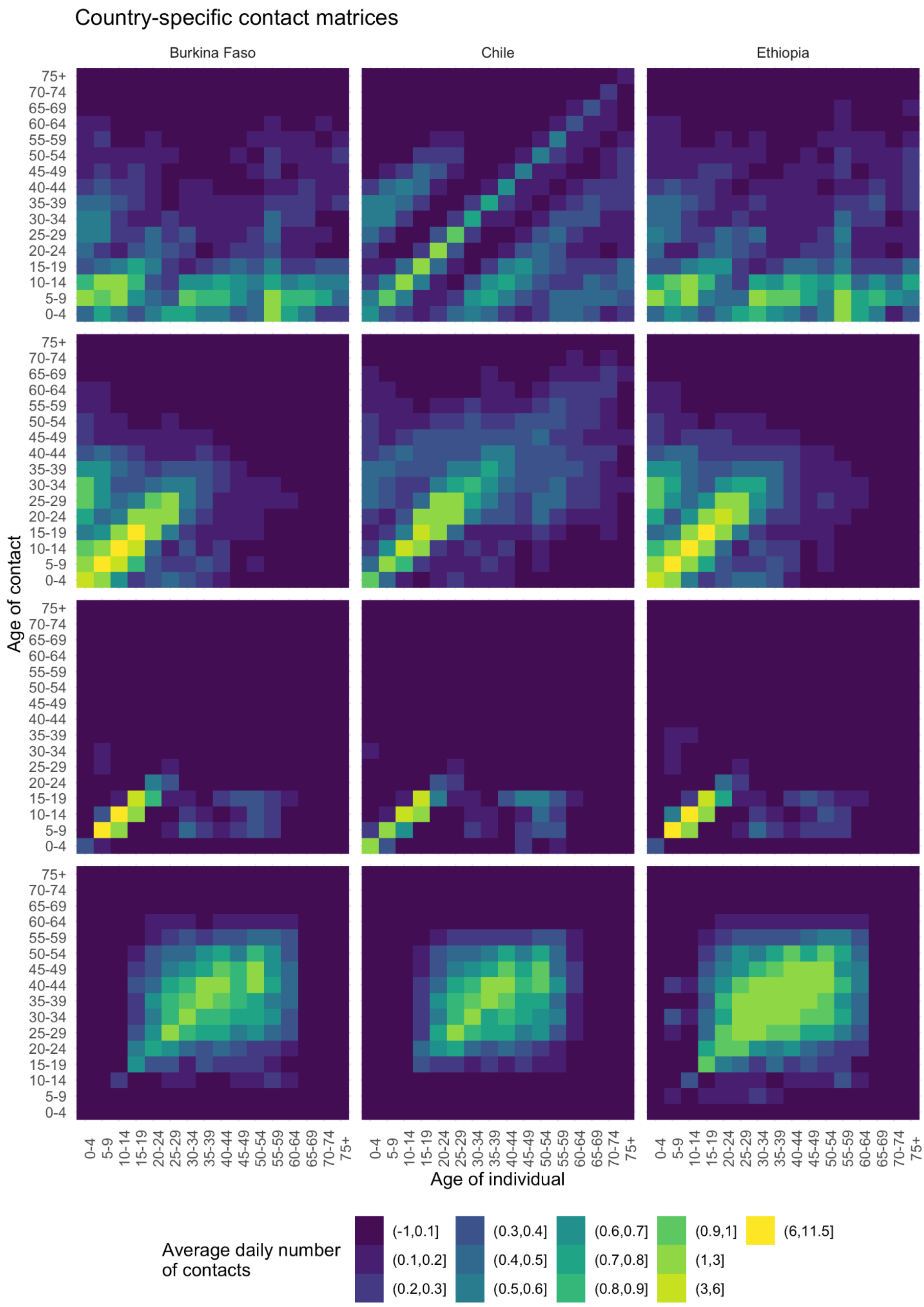

Supplementary Figure 2. Baseline age-specific contact matrices from Prem et al. (prior to assumed reduction in non-household contacts) used to parameterise the transmission model

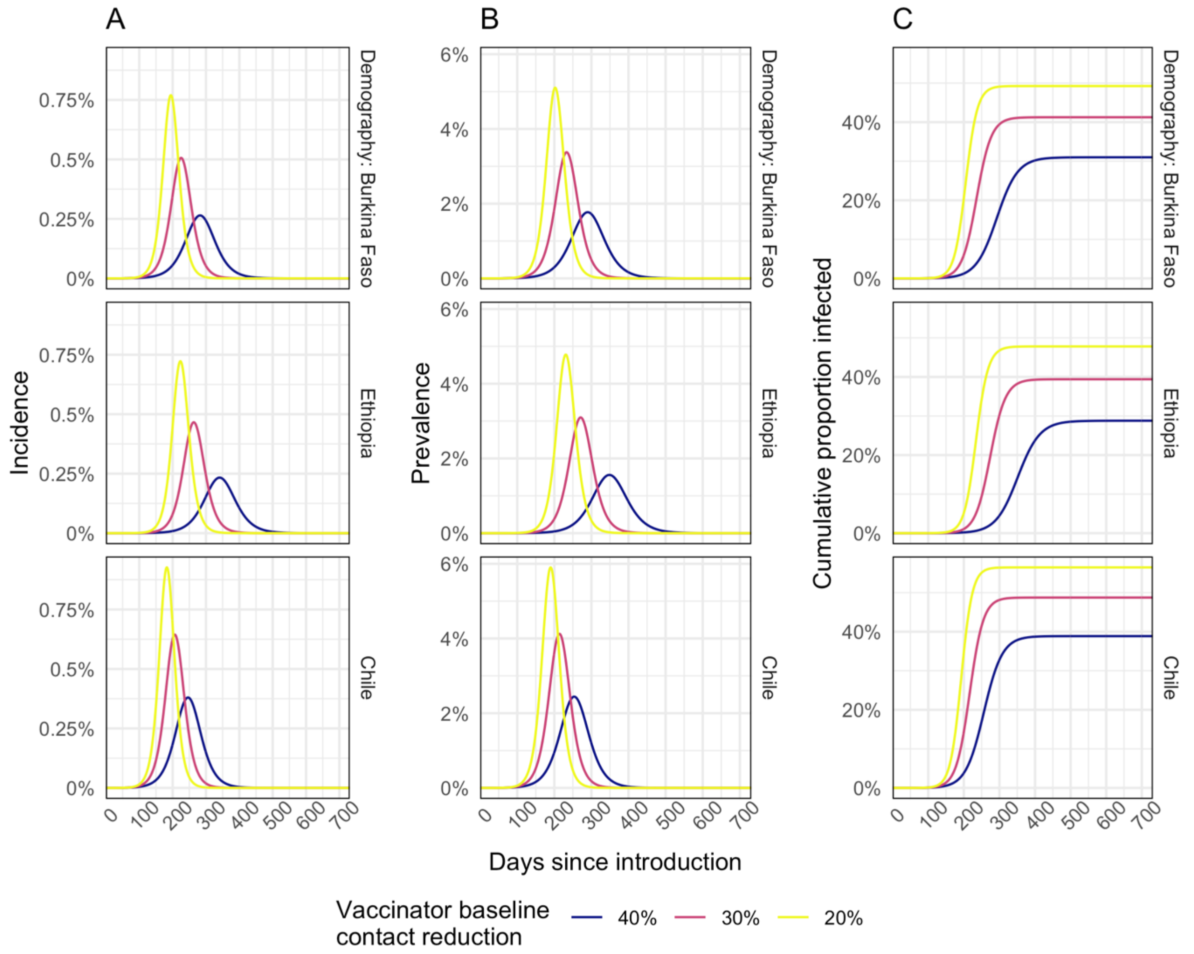

Supplementary Figure 3. Modelled incidence (A), prevalence (B) and cumulative proportion of individuals infected (C) for an epidemic with R_0 = 2 and assuming vaccinators experience different % reductions in non-household contacts compared to baseline.

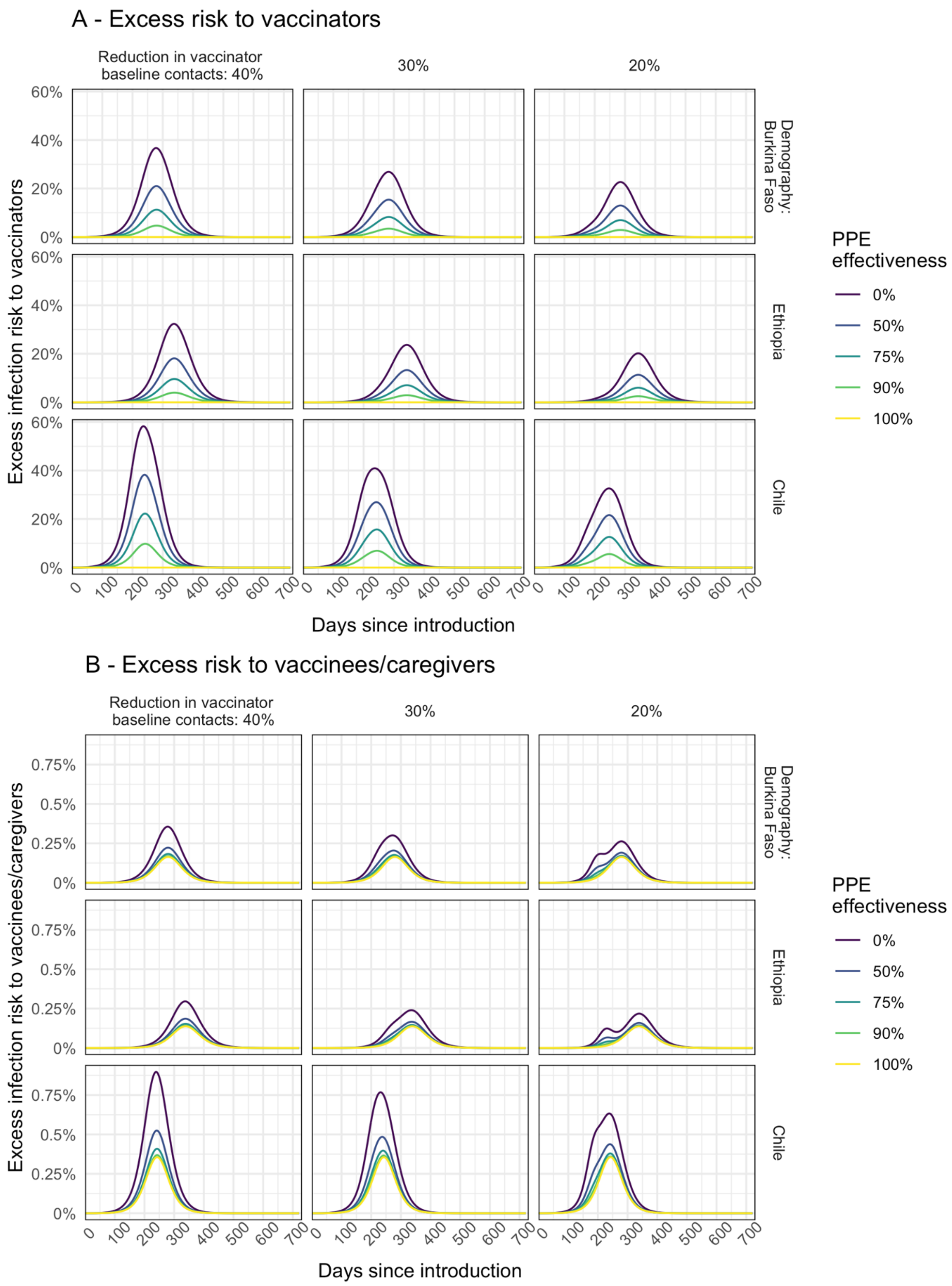

Supplementary Figure 4. Excess infection risk for vaccinators (A) and children and/or caregivers (B) for scenarios in which vaccinators are assumed to experience a smaller reduction in non-household contacts compared to the general population.

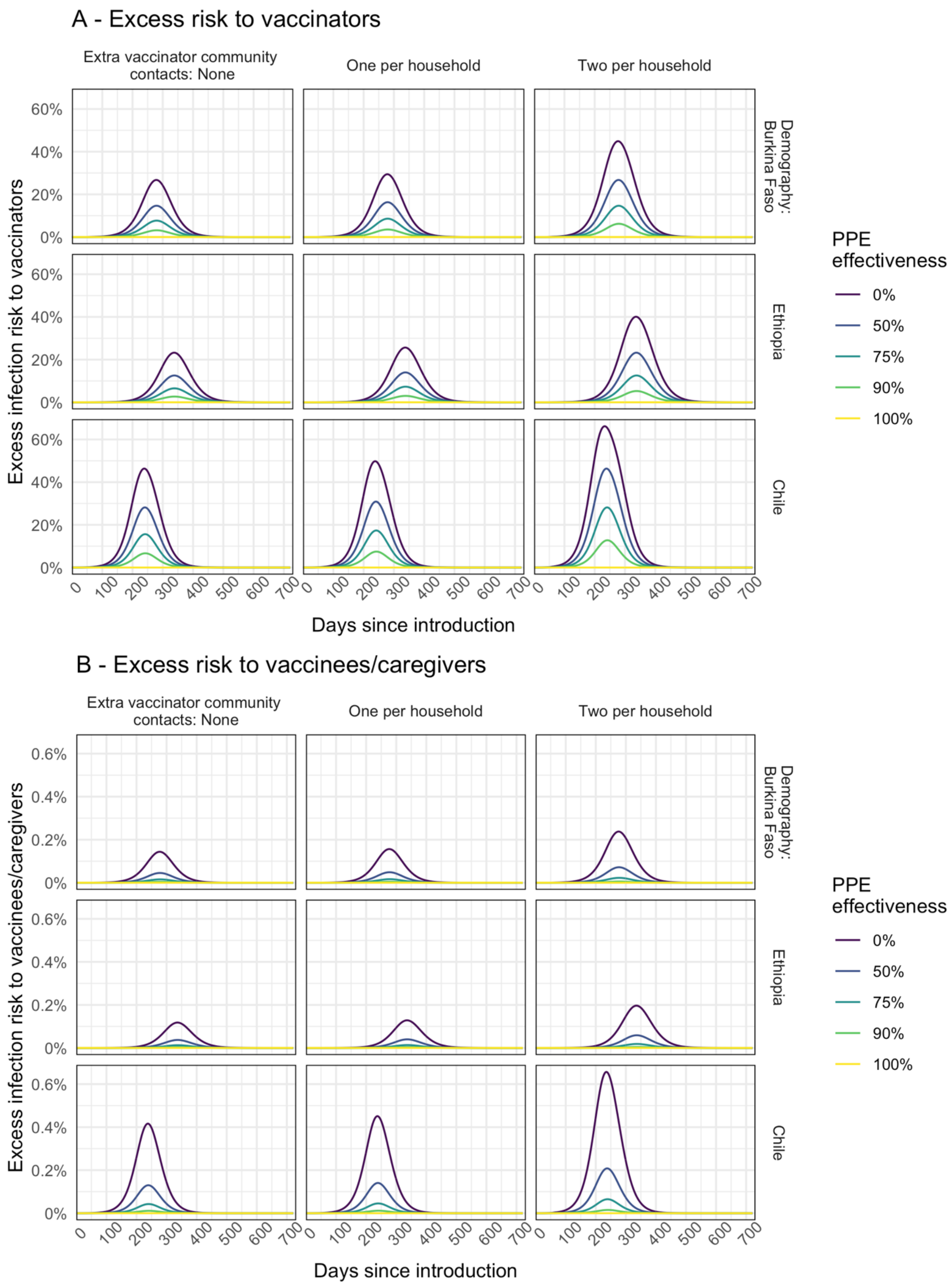

Supplementary Figure 5. Modelled excess risk of (A) vaccinators and (B) vaccinnees and/or caregivers becoming infected during house-to-house immunisation campaigns conducted at different times during the epidemic. Results are shown for epidemics modelled using R_0=2 and different assumptions about the number of additional community contacts made by vaccinators for each household visited. Line colour shows the impact of different levels of PPE effectiveness.
